# Correction: Decreased spinal inhibition leads to undiversified locomotor patterns

**DOI:** 10.1007/s00422-026-01040-w

**Published:** 2026-06-17

**Authors:** Myriam Lauren de Graaf, Heiko Wagner, Luis Mochizuki, Charlotte Le Mouel

**Affiliations:** 1 Department of Movement Science, University of Münster, Horstmarer Landweg 62B, 48149 Münster, Germany; 2 Otto Creutzfeldt Center for Cognitive and Behavioral Neuroscience, University of Münster, Fliednerstraße 21, 48149 Münster, Germany; 3 Center for Nonlinear Science (CeNoS), University of Münster, Corrensstraße 2, 48149 Münster, Germany; 4 School of Arts, Sciences and Humanities (EACH), University of São Paulo, Arlindo Bettio 1000, São Paulo, 03828-000 Brazil; 5 Institut Des Systèmes Intelligents et de Robotique (ISIR), Sorbonne Université, CNRS, 75005 Paris, France

**Correction to: Biological Cybernetics (2025) 119:12** https://doi.org/10.1007/s00422-025-01011-7

In Sect. 2.2, the sentence beginning ‘By averaging across different’, the notation ‘ϕ’ should have read ‘φ’.

In this article, in several equations the notation “ϕ” should have read “φ”. In addition, the notations “ϕ and φ” were swapped in the Eqs. 6 and 7. The corrected equations are provided below.

Equation 6:

$$ \left[ {\varphi^{2} } \right] = \, \int {Dz \cdot \phi^{2} \left( {\mu + \sqrt \Delta z} \right)} $$

Equation 7:

$$ \left[ \varphi \right] = \, \int {Dz \cdot \phi \left( {\mu + \sqrt \Delta z} \right)} $$

Equation 47:

$$ \tau \frac{d}{dt}\mu (t) = - \mu (t) + (m_{E} N_{E} + m_{I} N_{I} )\left[ {\varphi (t)} \right] $$

Equation 51:

$$ \tau \frac{d}{dt}\Delta = - \Delta (t) + (v_{E} N_{E} + v_{I} N_{I} )\left[ {\varphi^{2} (t)} \right] + v_{In} U(t)^{2} $$

Equation 52:

$$ \begin{aligned} \tau \frac{d}{dt}\mu (t) = & - \mu (t) + (m_{E} N_{E} + m_{I} N_{I} )\left[ {\varphi (t)} \right] \\ & = { \mathcal{N}}_{\mu } (\mu (t),\Delta (t)) \\ \end{aligned} $$

Equation 53:

$$ \begin{aligned} \tau \frac{d}{dt}\Delta = & - \Delta (t) + (v_{E} N_{E} + v_{I} N_{I} )\left[ {\varphi^{2} (t)} \right] + v_{In} U(t)^{2} \\ & = { \mathcal{N}}_{\Delta } (\mu (t),\Delta (t),U(t)) \\ \end{aligned} $$

Equation 55:

$$ \frac{{\partial { \mathcal{N}}_{\mu } }}{\partial \mu } = \, - 1 + (m_{E} N_{E} + m_{I} N_{I} )\frac{\partial }{\partial \mu }\left[ {\varphi (t)} \right] $$

Equation 56:

$$ \begin{aligned} \frac{\partial }{\partial \mu }\left[ {\varphi (t)} \right] = & \frac{\partial }{\partial \mu }\int D z\phi (\mu (t) + \sqrt {\Delta (t)} z) \\ & = \int D z\phi^{\prime}(\mu_{0} + \sqrt {\Delta_{0} } z) = \left[ {\varphi^{\prime \prime}} \right]_{0} \\ \end{aligned} $$

Equation 57:

$$ \frac{{\partial { \mathcal{N}}_{\mu } }}{\partial \Delta } = \, (m_{E} N_{E} + m_{I} N_{I} )\frac{\partial }{\partial \Delta }\left[ {\varphi (t)} \right] $$

Equation 58:

$$ \begin{aligned} \frac{\partial }{\partial \Delta }\left[ {\varphi (t)} \right] = & \frac{\partial }{\partial \Delta }\int D z\phi (\mu (t) + \sqrt {\Delta (t)} z) \\ & = \int D zz\frac{1}{{2\sqrt {\Delta_{0} } }}\phi^{\prime}(\mu_{0} + \sqrt {\Delta_{0} } z) \\ \end{aligned} $$

Equation 59:

$$ \frac{\partial }{\partial \Delta }\left[ {\varphi (t)} \right] = \frac{1}{{2\sqrt {\Delta_{0} } }}\int D z\sqrt {\Delta_{0} } \phi^{\prime\prime}(\mu_{0} + \sqrt {\Delta_{0} } z) = \frac{1}{2}\left[ {\varphi ^{\prime\prime }} \right]_{0} $$

Equation 60:

$$ \begin{aligned} \tau \frac{d}{dt}\mu (t) = & \delta \mu (t)\left( { - 1 + (m_{E} N_{E} + m_{I} N_{I} )\left[ {\varphi { \prime \prime}} \right]_{0} } \right) \\ & \quad + \delta \Delta (t)\frac{1}{2}(m_{E} N_{E} + m_{I} N_{I} )\left[ {\varphi ^{\prime\prime }} \right]_{0} \\ \end{aligned} $$

Equation 62:

$$ \frac{{\partial { \mathcal{N}}_{\Delta } }}{\partial \mu } = (v_{E} N_{E} + v_{I} N_{I} )\frac{\partial }{\partial \mu }\left[ {\varphi^{2} (t)} \right] $$

Equation 63:

$$ \begin{aligned}   \frac{\partial }{{\partial \mu }}\left[ {\varphi ^{2} (t)} \right] =  & \,\frac{\partial }{{\partial \mu }}\int D z\phi ^{2} (\mu (t) + \sqrt {\Delta (t)} z) \\     =  & \,\int D z2\phi (\mu _{0}  + \sqrt {\Delta _{0} } z)\phi ^{{\prime \prime }} (\mu _{0}  + \sqrt {\Delta _{0} } z) \\     =  & \,2\left[ {\varphi \varphi ^{{\prime \prime }} } \right]_{0}  \\  \end{aligned}  $$

Equation 64:

$$ \frac{{\partial { \mathcal{N}}_{\Delta } }}{\partial \Delta } = - 1 + (v_{E} N_{E} + v_{I} N_{I} )\frac{\partial }{\partial \Delta }\left[ {\varphi^{2} (t)} \right] $$

Equation 65:

$$ \begin{aligned} \frac{\partial }{\partial \Delta }\left[ {\varphi^{2} (t)} \right] = & \frac{\partial }{\partial \Delta }\int D z\phi^{2} (\mu (t) + \sqrt {\Delta (t)} z) \\ & = \int D z2\frac{z}{{2\sqrt {\Delta_{0} } }}\phi (\mu_{0} + \sqrt {\Delta_{0} } z)\phi^{\prime}(\mu_{0} + \sqrt {\Delta_{0} } z) \\ \end{aligned}$$

Equation 67:

$$ = \left[ {(\varphi^{ \prime\prime })^{2} } \right]_{0} + \left[ {\varphi \varphi ^{\prime\prime }} \right]_{0} $$

Equation 68:

$$ \begin{aligned} \tau \frac{d}{dt}\Delta = & (v_{E} N_{E} + v_{I} N_{I} )2\left[ {\varphi \varphi^{ \prime\prime }} \right]_{0} \delta \mu (t) \\ & \quad + \left( { - 1 + (v_{E} N_{E} + v_{I} N_{I} )\left( {\left[ {(\varphi^{ \prime\prime })^{2} } \right]_{0} + \left[ {\varphi \varphi ^{\prime\prime }} \right]_{0} )} \right)} \right)\delta \Delta (t) \\ \end{aligned}$$

Equation 69:

$$
\begin{aligned}
&\tau \frac{d}{dt}\left( {\begin{array}{*{20}c}
{\delta \mu } \\
{\delta \Delta } \\
\end{array} } \right) = - \left( {\begin{array}{*{20}c}
{\delta \mu } \\
{\delta \Delta } \\
\end{array} } \right) \\
&\quad + \left( {\begin{array}{*{20}c}
{(m_{E} N_{E} + m_{I} N_{I} )\left[ {\varphi^{\prime \prime}} \right]_{0} } &
{\frac{1}{2}(m_{E} N_{E} + m_{I} N_{I} )\left[ {\varphi^{ \prime\prime }} \right]_{0} } \\
{2(v_{E} N_{E} + v_{I} N_{I} )\left[ {\varphi \varphi^{ \prime\prime }} \right]_{0} } &
{(v_{E} N_{E} + v_{I} N_{I} )\left( {\left[ {(\varphi^{ \prime\prime })^{2} } \right]_{0} + \left[ {\varphi \varphi ^{\prime\prime }} \right]_{0} )} \right)} \\
\end{array} } \right) \\
&\quad\quad \left( {\begin{array}{*{20}c}
{\delta \mu } \\
{\delta \Delta } \\
\end{array} } \right)
\end{aligned}
$$

Equation 70:

$$
\begin{aligned}{\boldsymbol{M}} = \left( {\begin{array}{*{20}c} {(m_{E} N_{E} + m_{I} N_{I} )\left[ {\varphi^{ \prime\prime }} \right]_{0} } & {\frac{1}{2}(m_{E} N_{E} + m_{I} N_{I} )\left[ {\varphi^{ \prime\prime }} \right]_{0} } \\ {2(v_{E} N_{E} + v_{I} N_{I} )\left[ {\varphi \varphi^{ \prime\prime }} \right]_{0} } & {(v_{E} N_{E} + v_{I} N_{I} )\left( {\left[ {(\varphi^{\prime})^{2} } \right]_{0} + \left[ {\varphi \varphi^{ \prime\prime }} \right]_{0} )} \right)} \\ \end{array} } \right)\end{aligned}
$$

Equation 71:

$$ a = (m_{E} N_{E} + m_{I} N_{I} )\left[ {\varphi^{ \prime\prime }} \right]_{0} $$

Equation 72:

$$ b = \, \frac{1}{2}(m_{E} N_{E} + m_{I} N_{I} )\left[ {\varphi^{ \prime \prime }} \right]_{0} $$

Equation 73:

$$ c = 2(v_{E} N_{E} + v_{I} N_{I} )\left[ {\varphi \varphi^{ \prime\prime }} \right]_{0} $$

Equation 74:

$$ d = \, (v_{E} N_{E} + v_{I} N_{I} )\left( {\left[ {(\varphi^{ \prime \prime})^{2} } \right]_{0} + \left[ {\varphi \varphi { \prime \prime}} \right]_{0} )} \right) $$

The original article has been corrected.

